# Adiponectin Isoforms and Leptin Impact on Rheumatoid Adipose Mesenchymal Stem Cells Function

**DOI:** 10.1155/2016/6532860

**Published:** 2015-11-22

**Authors:** Urszula Skalska, Ewa Kontny

**Affiliations:** Department of Immunology and Pathophysiology, Institute of Rheumatology, Ul. Spartańska 1, 02-637 Warsaw, Poland

## Abstract

Adiponectin and leptin have recently emerged as potential risk factors in rheumatoid arthritis (RA) pathogenesis. In this study we evaluated the effects of adiponectin and leptin on immunomodulatory function of adipose mesenchymal stem cells (ASCs) derived from infrapatellar fat pad of RA patients. ASCs were stimulated with leptin, low molecular weight (LMW) and high/middle molecular weight (HMW/MMW) adiponectin isoforms. The secretory activity of ASCs and their effect on rheumatoid synovial fibroblasts (RA-FLS) and peripheral blood mononuclear cells (PBMCs) from healthy donors have been analysed. RA-ASCs secreted spontaneously TGF*β*, IL-6, IL-1Ra, PGE2, IL-8, and VEGF. Secretion of all these factors was considerably upregulated by HMW/MMW adiponectin, but not by LMW adiponectin and leptin. Stimulation with HMW/MMW adiponectin partially abolished proproliferative effect of ASC-derived soluble factors on RA-FLS but did not affect IL-6 secretion in FLS cultures. ASCs pretreated with HMW/MMW adiponectin maintained their anti-inflammatory function towards PBMCs, which was manifested by moderate PBMCs proliferation inhibition and IL-10 secretion induction. We have proved that HMW/MMW adiponectin stimulates secretory potential of rheumatoid ASCs but does not exert strong impact on ASCs function towards RA-FLS and PBMCs.

## 1. Introduction

Adiponectin and leptin have been recently considered to be important factors in rheumatoid arthritis (RA) pathogenesis [1]. Both these adipokines play well known metabolic role but also exert impact on immune system. Leptin is a proinflammatory factor stimulating innate and acquired immune response and its concentration increases during infection and inflammation. Adiponectin exerts opposite effects to leptin; however, many reports about its action are conflicting, probably due to the existence of several isoforms of this adipokine [2]. In plasma, adiponectin circulates in four isoforms: low molecular weight (LMW) isoform, middle molecular weight (MMW) isoform, high molecular weight (HMW) isoform, and globular isoform, exhibiting different effects on immune system. HMW isoform is thought to possess proinflammatory activity, whereas LMW isoform is thought to possess anti-inflammatory activity [2].

RA is an autoimmune disease characterized by synovial fibroblasts (FLS) hyperproliferation, overactivation of lymphocytes, synovial membrane inflammation, cartilage destruction, and bone erosion. In the rheumatoid joint, apart from synovium, cartilage, and bone, fat pads are affected by inflammatory process [3]. Intra-articular adipose tissue is a local source of proinflammatory adipokines [4] as well as of adipose mesenchymal stem cells (ASCs) possessing immunoregulatory and regenerative properties. These cells are very promising in terms of cell therapy of autoimmune and degenerative diseases [5]. Mesenchymal stem cells (MSCs) have been shown to suppress T cells activation and to induce T regulatory cells (Tregs) generation. Their immunoregulatory effects are mediated by various factors, for example, transforming growth factor *β* (TGF*β*), interleukin 6 (IL-6), interleukin 10 (IL-10), interleukin 1 receptor antagonist (IL-1Ra), and prostaglandin E2 (PGE2) [6]. Because MSCs immunosuppressive activity is dependent on stimuli provided by local environment [6], we hypothesize that adipocytokines present in rheumatoid joint may affect ASCs function. The aim of this study was to evaluate the effects of leptin and adiponectin isoforms on function of ASCs derived from rheumatoid infrapatellar fat pad (IPFP).

## 2. Materials and Methods

### 2.1. ASCs Donors and ASCs Treatment

Infrapatellar fat pads (IPFPs) were obtained from 29 RA patients undergoing total knee joint replacement surgery. All patients were selected from the Rheumoortopaedic Clinic of the Institute of Rheumatology in Warsaw and gave their written informed consent according to the Declaration of Helsinki. The study was approved by the Institute of Rheumatology Ethics Committee. ASCs were isolated and cultured in medium composed of DMEM/F12, 10% fetal calf serum, and antibiotics. Cells showed differentiation capacity to the chondrogenic and osteogenic lineages and had the CD105^+^CD90^+^CD73^+^CD45^−^CD34^+/−^CD19^−^CD14^−^ phenotype. ASCs (2 × 10^4^/well/1 mL) at passages 3–5 were seeded in culture medium and were treated or not with 10 ng/mL of recombinant human (rh) leptin (PeproTech, London, UK), 10 *μ*g/mL of rhLMW adiponectin, or 10 *μ*g/mL of rhHMW/MMW adiponectin (BioVendor, Brno, Czech Republic). Concentrations of abovementioned adipocytokines were selected on basis of their concentration in serum of RA patients [7].

### 2.2. Immunoenzymatic Assays

After 24 h of stimulation, culture supernatants (SNs) from ASCs were harvested. Concentrations of the following factors in SNs were measured by specific ELISAs: IL-6, IL-8 (using own procedures as previously described [8]), TGF*β*, VEGF, IL-1Ra (DuoSet kits, R&D Systems, Minneapolis, MN, USA), and PGE2 (Parameter kit, R&D Systems).

### 2.3. Conditioned Media Preparation

For ASC-conditioned media (CM) preparation, ASCs were seeded and cultured for 24 h with addition of adipocytokines as described above; then, medium in stimulated ASCs cultures was replaced with the fresh one, deprived of any stimuli. After additional 24 h of culture, SNs (in volume of 900 *μ*L) were harvested and used for RA-FLS cultures.

### 2.4. Rheumatoid FLS Treatment

Rheumatoid synovial fibroblasts (RA-FLS), isolated from rheumatoid synovial membranes and cultured as described previously [8], were seeded (2 × 10^4^/well/1 mL) in CM or cocultured with ASCs (2 × 10^4^/well/1 mL). SNs from cocultures were harvested after 48 h, whereas in CM-treated cells the medium was replaced with the fresh one after 24 h and harvested after additional 24 h. Concentration of IL-6 was measured in SNs as described above. For proliferation assay, RA-FLS (2 × 10^4^/well/0.1 mL) were cultured in CM or with ASCs (1 × 10^4^/well/0.1 mL) for 48 h. Proliferation was evaluated by bromodeoxyuridine (BrdU) cell proliferation assay (Millipore Corporation, Billerica, MA, USA).

### 2.5. Peripheral Blood Mononuclear Cells Treatment

Peripheral blood mononuclear cells (PBMCs), isolated from buffy coats of healthy male donors according to routinely applied procedure using Ficoll-Paque (GE Healthcare, Uppsala, Sweden), were treated or not with 2.5 *μ*g/mL of phytohaemagglutinin (PHA) and then cultured (4 × 10^5^/well/1 mL) for 24 h with ASCs (2 × 10^4^/well/1 mL). SNs from cocultures were harvested after 24 h and concentration of IL-10 was measured in SNs (Ready-SET-Go kit, eBioscience, San Diego, CA, USA). For proliferation assay, PBMCs (1 × 10^5^/well/0.1 mL) were seeded onto ASCs (1 × 10^4^/well/0.1 mL) and cultured for 72 h. PBMCs proliferation was evaluated using ^3^H-thymidine incorporation assay.

### 2.6. Statistical Analysis

Statistical analysis was performed using STATISTICA 10.0 software (Stat Soft Inc., Tulsa, OK, USA). Obtained data was not normally distributed, as assessed by Shapiro-Wilk test. The differences between samples were evaluated using the nonparametric Wilcoxon signed-rank test. All results are shown as arithmetic mean and standard error of a mean. Differences were considered statistically significant for ^*∗*^ *p* < 0.05, ^*∗∗*/##^ *p* < 0.01, and ^*∗∗∗*^ *p* < 0.001.

## 3. Results

**Figure 1:**
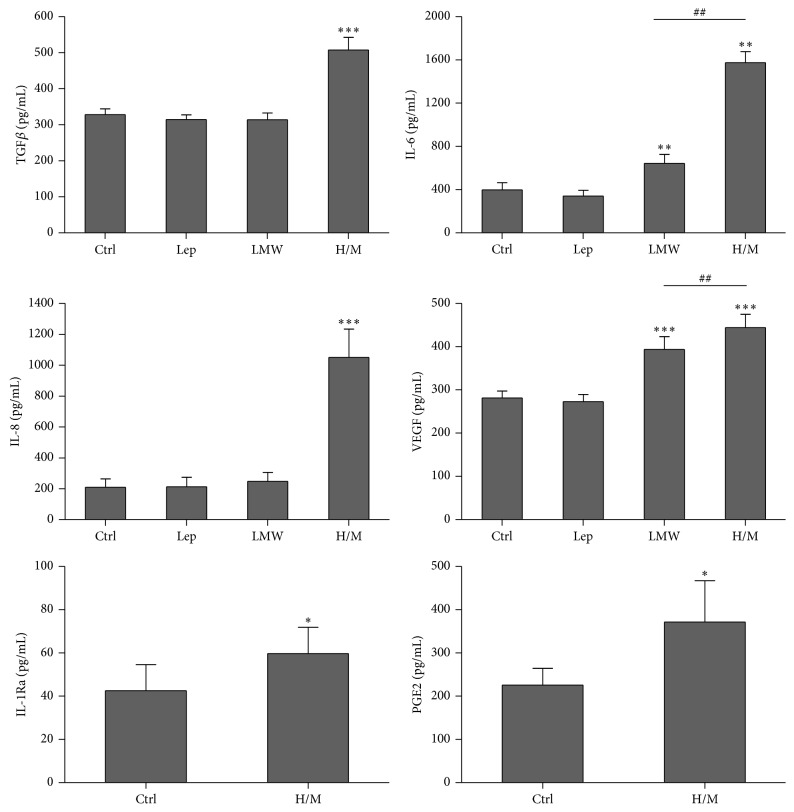
Adipokines effects on secretory activity of ASCs derived from rheumatoid infrapatellar fat pad. Unstimulated (Ctrl) or stimulated with 10 ng/mL leptin (Lep), 10 *μ*g/mL of LMW adiponectin, or 10 *μ*g/mL of HMW/MMW adiponectin ASCs from RA patients were cultured for 24 h. Concentrations of select factors were measured in supernatants collected from ASCs cultures using specific ELISA tests (*n* = 14–18). Asterisks (*∗*) indicate statistically significant differences* versus* Ctrl; hash marks (#) indicate statistically significant differences between indicated data. ^*∗*^ *p* < 0.05, ^*∗∗*/##^ *p* < 0.01, and ^*∗∗∗*^ *p* < 0.001.

Immunomodulatory properties of ASCs are associated with several soluble factors secretion [6]. As depicted in Figure 1, RA-ASCs secreted TGF*β*, IL-6, IL-1Ra, and PGE2 as well as IL-8 and VEGF which are related to angiogenesis process. Importantly, HMW/MMW adiponectin enhanced secretion of all factors, whereas LMW adiponectin and leptin exerted weaker or no effect, respectively (Figure 1). Given that among applied adipokines only HMW/MMW adiponectin affected ASCs secretory profile, we decided to test if HMW/MMW adiponectin-stimulated ASCs exert any effects on RA-FLS and PBMCs from healthy donors.

**Figure 2:**
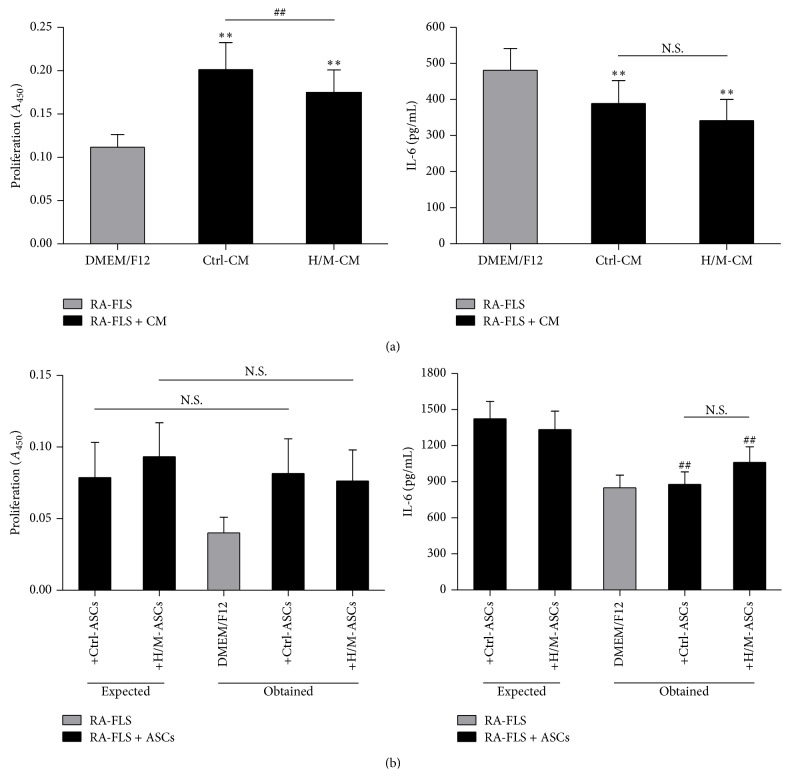
Effects of unstimulated and HMW/MMW adiponectin-stimulated RA-ASCs on RA-FLS function. RA-FLS were cultured either in conditioned media (CM) collected from RA-ASCs (a) or in the presence of RA-ASCs (b). For coculture experiments, unstimulated ASCs (+Ctrl-ASCs) or ASCs stimulated with 10 *μ*g/mL of HMW/MMW adiponectin (+H/M-ASCs) were used, whereas CM from respective cultures of unstimulated (Ctrl-CM) and stimulated (H/M-CM) ASCs were added to FLS. RA-FLS cultured separately (DMEM/F12) served as a control. Proliferation of cells was determined by BrdU incorporation assay. Absorbency of samples was measured at 450 nm (*A*_450_). IL-6 was detected by specific ELISA test. (a) The effects of ASC-CM on RA-FLS proliferation (*n* = 14) and IL-6 secretion (*n* = 17). (b) Proliferation (*n* = 6) and IL-6 (*n* = 9) secretion in ASCs-FLS cocultures. Expected: the sum of the values obtained for separate FLS and ASCs cultures; obtained: real values stated in the cocultures. Asterisks (*∗*) indicate statistically significant differences* versus* DMEM/F12; hash marks (#) indicate statistically significant differences between indicated data or between expected and obtained values; ^*∗∗*/##^ *p* < 0.01; N.S.: nonsignificant.

ASC-conditioned media (CM) contributed to upregulated proliferation of RA-FLS, which has already been reported by our group [9]. This effect was partially abolished by ASCs pretreatment with HMW/MMW adiponectin (Figure 2(a)), but not with LMW isoform or leptin (own data, not shown). Interestingly, proliferation of cells in cocultures stayed unchanged (Figure 2(b)). Both CM from ASCs and ASCs presence caused modest downregulation of IL-6 production, but it was independent of ASCs prestimulation. Upon treatment with CM from HMW/MMW adiponectin-stimulated ASCs, IL-6 secretion was slightly decreased comparing to CM from unstimulated ASCs; however, this difference did not reach statistical significance (Figure 2). LMW adiponectin- or leptin-treated ASCs did not exert any impact on IL-6 production in FLS cultures (own data, not shown).

**Figure 3:**
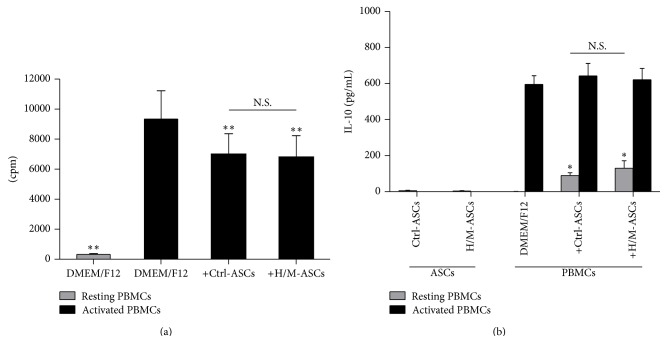
Effects of unstimulated and HMW/MMW adiponectin-stimulated RA-ASCs on proliferation of PBMCs from healthy donors and IL-10 secretion by these cells. PBMCs either were prestimulated with 2.5 *μ*g/mL of phytohaemagglutinin (activated PBMCs) or were left untreated (resting PBMCs) and then cultured in the presence of unstimulated ASCs (+Ctrl-ASCs) or ASCs stimulated with 10 *μ*g/mL of HMW/MMW adiponectin (+H/M-ASCs). Resting and activated PBMCs cultured separately in DMEM/F12 medium served as controls (DMEM/F12). Proliferation of cells was determined by ^3^H-thymidine incorporation assay. Radioactivity of samples is expressed in counts per minute (cpm). Concentration of IL-10 was measured in culture supernatants by specific ELISA test. (a) Proliferation of PBMCs cocultured in the presence of ASCs (*n* = 9). (b) IL-10 secretion in ASCs-PBMCs cocultures (*n* = 6). Asterisks (*∗*) indicate statistically significant differences* versus* activated (a) or resting (b) PBMCs cultured separately; ^*∗*^ *p* < 0.05; ^*∗∗*^ *p* < 0.01; N.S.: nonsignificant.

Despite the strong impact of HMW/MMW adiponectin on secretory potential of ASCs, this adipokine did not affect ASCs immunosuppressive potential against PHA-activated PBMCs from healthy donors. Proliferation of PBMCs was moderately inhibited by the presence of unstimulated ASCs, but pretreatment of ASCs with HMW/MMW adiponectin did not alter their antiproliferative effect (Figure 3(a)). Similarly, induction of anti-inflammatory IL-10 in resting PBMCs by ASCs was not further affected by prestimulation of ASCs with HMW/MMW adiponectin (Figure 3(b)).

## 4. Discussion

In this* in vitro *study, we have analysed the impact of select adipokines on function of human RA-ASCs derived from intra-articularly localized infrapatellar fat pad. Previously, we have demonstrated that RA-ASCs express genes coding for adiponectin (AdipoR1 and AdipoR2) and leptin (ObRb) receptors [10]. Here, we proved that HMW/MMW adiponectin acts as a strong stimulator of ASCs secretory activity, whereas leptin and LMW adiponectin do not exert considerable effects on ASCs. It is worth noting that some factors, such as IL-6, TGF*β*, and PGE_2_, responsible for immunosuppressive ASCs function [6] might be detrimental in RA pathogenesis [11, 12]. In this view, observed in this study, HMW/MMW adiponectin-induced IL-6, TGF*β*, and PGE_2_ secretion increase may be recognized as an intensification of ASCs anti-inflammatory activity and also as a contribution of these cells to the inflammation in the rheumatoid joint. Moreover, upregulation of IL-8 and VEGF, important proangiogenic factors associated with arthritis, seems to confirm this suggestion.

Studies about adiponectin isoforms effects on MSCs are not available; however, it is known that HMW/MMW adiponectin strongly upregulates secretion of cytokines and chemokines by RA-FLS, while LMW adiponectin does not have this impact, which was described independently by two groups [7, 13]. Moreover, HMW/MMW adiponectin was reported to be less potent in stimulating FLS from osteoarthritis patients [13]. Together with present results it shows that HMW adiponectin, but not LMW adiponectin and leptin, strongly affects rheumatoid cells and suggests this isoform contribution to RA pathogenesis.

Surprisingly, in spite of stimulating effect of HMW/MMW adiponectin on RA-ASCs secretory activity (Figure 1), we did not demonstrate that ASCs treated with this adipokine modify significantly their functions towards other cells. To evaluate HMW/MMW adiponectin impact on ASCs function we performed* in vitro* cocultures or cultures in ASC-conditioned media with FLS from rheumatoid patients and PBMCs from healthy donors. We aimed for* in vitro* setting in which only ASCs would derive from pathologic environment and responder cells would not be affected by inflammatory process. Nonetheless, in case of FLS, it was not possible due to ethical reasons.

In the course of RA, the synovial membrane inflammation develops and persists; FLS are overactivated, proliferate excessively, and produce proinflammatory mediators and tissues degrading enzymes [14]. We observed that soluble factors released by RA-ASCs exert proproliferative effects on RA-FLS, but it was partially abolished when ASCs were prestimulated with HMW/MMW adiponectin. Possibly, it was due to antiproliferative factors released by ASCs upon HMW/MMW adiponectin stimulation, for example, IL-6, TGF*β*, and PGE2 [6]. Interestingly, the lack of cell proliferation increase in coculture may indicate cell contact-dependent reciprocal inhibition of trophic factors production.

Interleukin 6 is associated with chronic inflammatory response in RA [12]. We have already shown that unstimulated ASCs moderately decreased IL-6 release in RA-FLS culture [9]. Here, we demonstrated that HMW/MMW adiponectin-pretreated ASCs exerted similar effect. Thus, despite postulated proinflammatory role of HMW adiponectin, we did not observe that this adipokine promotes proinflammatory ASCs function towards RA-FLS in respect of IL-6 secretion. This is in contrast to TNF, which, as we have recently reported, triggers proinflammatory capabilities of ASCs leading to upregulation of IL-6 and metalloproteinase 3 release by RA-FLS [9].

In the rheumatoid joint, mononuclear lymphoid cells infiltrate into synovial membrane and form ectopic lymphoid tissue. T lymphocytes activated in germinal centers of ectopic lymphoid follicles proliferate excessively and contribute to the chronic inflammation by proinflammatory cytokines secretion [15]. In our study, unstimulated ASCs limited proliferation of PHA-activated PBMCs and induced IL-10 production by resting PBMCs. Because ASCs pretreated with HMW/MMW adiponectin did not alter their impact on PBMCs (Figure 3), it is clear that this adipokine does not affect ASCs immunosuppressive activity towards lymphoid cells.

It must be underlined in this section that present study has several limitations. Due to ethical reasons, we were unable to obtain the IPFP and synovial membrane from healthy donors. Thus, we could not provide responder FLS from healthy individuals and our analysis was limited to unstimulated* versus* stimulated rheumatoid ASCs. Comparing IPFP-derived ASCs with ASCs from subcutaneous adipose tissue (obtained from lipoaspirates) would be also questionable, as it is known that different adipose tissue depots have distinct secretory potential possibly influencing resident cells, and ASCs derived from different fat source exhibit diverse characteristics (i.e., proliferative and regenerative potential) [16–18].

In conclusion, we demonstrated that HMW/MMW adiponectin, but not LMW adiponectin and leptin, stimulates considerably secretory potential of rheumatoid ASCs suggesting its contribution to RA pathogenesis. However, regardless of postulated proinflammatory role of HMW adiponectin, we did not prove that this adipokine induces proinflammatory ASCs activity towards RA-FLS and PBMCs.

## References

[1] Abella V., Scotece M., Conde J. (2014). Adipokines, metabolic syndrome and rheumatic diseases. *Journal of Immunology Research*.

[2] Krysiak R., Handzlik-Orlik G., Okopien B. (2012). The role of adipokines in connective tissue diseases. *European Journal of Nutrition*.

[3] Caulfield J. P., Hein A., Helfgott S. M., Brahn E., Dynesius-Trentham R. A., Trentham D. E. (1988). Intraarticular injection of arthritogenic factor causes mast cell degranulation, inflammation, fat necrosis, and synovial hyperplasia. *Laboratory Investigation*.

[4] Kontny E., Plebanczyk M., Lisowska B., Olszewska M., Maldyk P., Maslinski W. (2012). Comparison of rheumatoid articular adipose and synovial tissue reactivity to proinflammatory stimuli: contribution to adipocytokine network. *Annals of the Rheumatic Diseases*.

[5] Kim N., Cho S.-G. (2013). Clinical applications of mesenchymal stem cells. *Korean Journal of Internal Medicine*.

[6] Ma S., Xie N., Li W., Yuan B., Shi Y., Wang Y. (2014). Immunobiology of mesenchymal stem cells. *Cell Death and Differentiation*.

[7] Kontny E., Janicka I., Skalska U., Maśliński W. (2015). The effect of multimeric adiponectin isoforms and leptin on the function of rheumatoid fibroblast-like synoviocytes. *Scandinavian Journal of Rheumatology*.

[8] Kontny E., Grabowska A., Kowalczewski J. (1999). Taurine chloramine inhibition of cell proliferation and cytokine production by rheumatoid arthritis fibroblast-like synoviocytes. *Arthritis & Rheumatism*.

[9] Skalska U., Kontny E. (2015). Inflammatory microenvironment of rheumatoid and osteoarthritic joint affects immunomodulatory activity of adipose-derived mesenchymal stem cells. *Annals of the Rheumatic Diseases*.

[10] Skalska U., Kontny E. (2013). Comparison of phenotype, chondrogenic and osteogenic potential of rheumatoid mesenchymal stem cells derived from articular and subcutaneous adipose tissue—the role of adipocytokines. *Central-European Journal of Immunology*.

[11] Martel-Pelletier J., Pelletier J.-P., Fahmi H. (2003). Cyclooxygenase-2 and prostaglandins in articular tissues. *Seminars in Arthritis and Rheumatism*.

[12] Niu X., Chen G. (2014). Clinical biomarkers and pathogenic-related cytokines in rheumatoid arthritis. *Journal of Immunology Research*.

[13] Frommer K. W., Schaffler A., Buchler C. (2012). Adiponectin isoforms: a potential therapeutic target in rheumatoid arthritis?. *Annals of the Rheumatic Diseases*.

[14] Boissier M.-C., Semerano L., Challal S., Saidenberg-Kermanac'h N., Falgarone G. (2012). Rheumatoid arthritis: from autoimmunity to synovitis and joint destruction. *Journal of Autoimmunity*.

[15] Manzo A., Pitzalis C. (2007). Lymphoid tissue reactions in rheumatoid arthritis. *Autoimmunity Reviews*.

[16] Kontny E., Prochorec-Sobieszek M. (2013). Articular adipose tissue resident macrophages in rheumatoid arthritis patients: potential contribution to local abnormalities. *Rheumatology*.

[17] Mochizuki T., Muneta T., Sakaguchi Y. (2006). Higher chondrogenic potential of fibrous synovium- and adipose synovium-derived cells compared with subcutaneous fat-derived cells: distinguishing properties of mesenchymal stem cells in humans. *Arthritis and Rheumatism*.

[18] Peptan I. A., Hong L., Mao J. J. (2006). Comparison of osteogenic potentials of visceral and subcutaneous adipose-derived cells of rabbits. *Plastic and Reconstructive Surgery*.

